# Supplementary figures and images for: Anti-prion drugs do not improve survival in novel knock-in models of inherited prion disease
Source: PLoS Pathog. 2024 Apr 1;20(4):e1012087. doi: 10.1371/journal.ppat.1012087 (PMC10984475; doi:10.1371/journal.ppat.1012087)

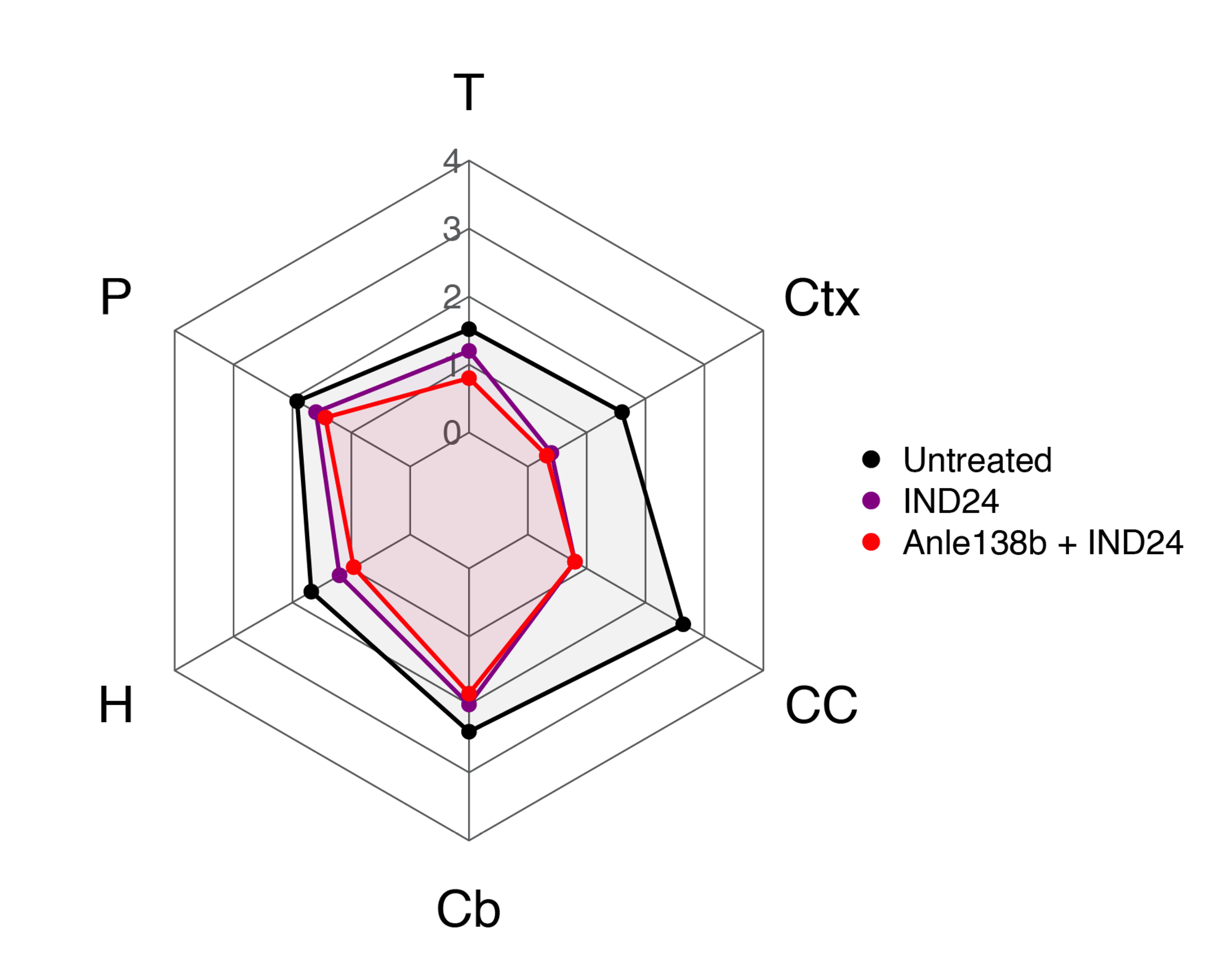

Supplement: S1 Fig — Mean GFAP immunohistochemistry scores (n = 2–7) in various brain regions of mice treated with various drug regimens, as specified in the legends. T = Thalamus, Ctx = cerebral cortex, CC = corpus callosum, Cb = cerebellum, H = hippocampus, P = pons. (TIF) [file ppat.1012087.s001.tif]

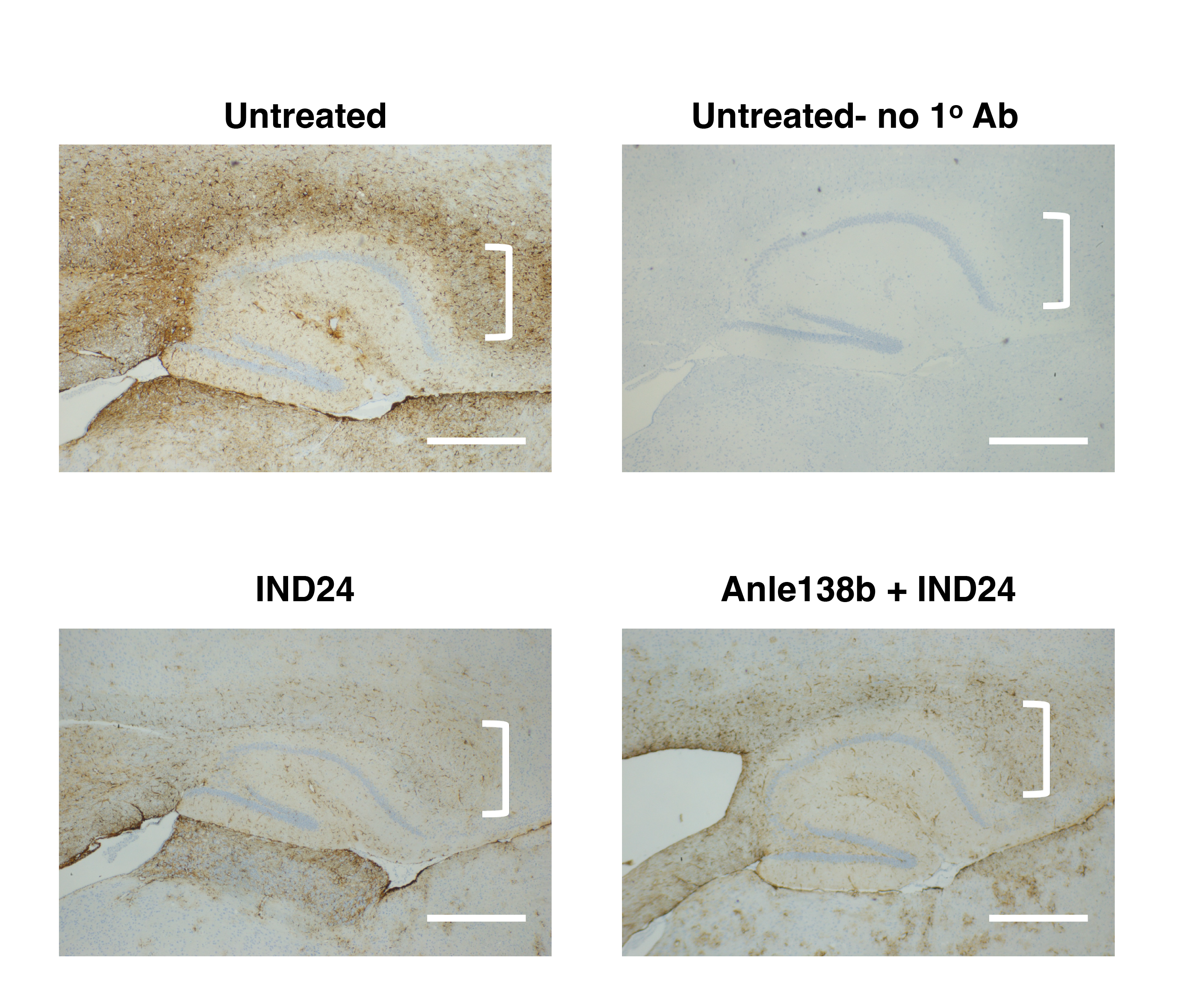

Supplement: S2 Fig — Representative microscopic images of brain sections of kiBVIE200K mice treated with various drug regimens, as specified, stained with hematoxylin and eosin. Square brackets indicate the location of the corpus callosum (in between the cerebral cortex and hippocampus). Horizontal scale bar = 500 μm. (TIF) [file ppat.1012087.s002.tif]
