# Supplementary material for: Anti-prion drugs do not improve survival in novel knock-in models of inherited prion disease
Source: PLoS Pathog. 2024 Apr 1;20(4):e1012087. doi: 10.1371/journal.ppat.1012087 (PMC10984475; doi:10.1371/journal.ppat.1012087)
Supplement: S1 Text — (DOC) [file ppat.1012087.s003.doc]

**Supplemental Information for:**

**Anti-prion drugs do not improve survival in knock-in models of inherited prion disease**

Daniel J. Walsh, Judy R. Rees, Surabhi Mehra, Matthew E.C. Bourkas, Lech Kaczmarczyk, Erica Stuart, Walker S. Jackson, Joel C. Watts, and Surachai Supattapone

**Figs A-F: Mutant PrP^Sc^ molecules in brains of kiBVI^D178N^ and kiBVI^E200K^ mice.** Western blots showing insoluble and thermolysin-resistant PrPSc molecules in brain homogenates of knock-in kiBVI^D178N^ and kiBVI^E200K^ mice treated with various drug regimens, as indicated. Samples were either treated with thermolysin (TL) or water, as indicated. All samples were centrifuged to collect insoluble PrP. Blots were probed with either anti-PrP mAb 27-33 or mAb EP1802Y, as indicated. **A:** untreated mice. **B:** mice treated with Anle138b. **C:** mice treated with IND24. **D:** mice treated with a combination regimen of Anle138b + IND24. **E:** mice treated with PSCMA. **F:** mice treated with Metolose.
